# Social Media in the Urology Practice | Opinion: YES

**DOI:** 10.1590/S1677-5538.IBJU.2019.05.03

**Published:** 2019-01-29

**Authors:** Mateus Cosentino Bellote, Hegel Trujillo Santamaria, Marcela Pelayo-Nieto, ES Heman Prasad, Nariman Gadzhiev, Kalyan Gudaru

**Affiliations:** 1 Departmentamento de Urologia Universidade Federal do Paraná Curitiba PR Brasil Departmentamento de Urologia, Universidade Federal do Paraná - UFPR, Curitiba, PR, Brasil;; 2 Instituto Mexicano del Seguro Social Centro Medico Nacional Unidad Medica De Alta especialidade México Instituto Mexicano del Seguro Social. Centro Medico Nacional, Unidad Medica De Alta especialidade No.14 “Lic. Adolfo Ruiz Cortines” Veracruz, México;; 3 Centro Médico Puerta de Hierro Zapopan Jalisco Mexico Centro Médico Puerta de Hierro, Zapopan, Jalisco, Mexico;; 4 CIHSR Referral Hospital Nagaland India CIHSR Referral Hospital, Nagaland, India;; 5 Departament of Endourological Pavlov First Saint Petersburg Medical University Russia Departament of Endourological, Pavlov First Saint Petersburg Medical University, Russia;; 6 Department of Urology Sri Venkateswara Institute Of Medical Sciences Tirupati Andhra Pradesh India Department of Urology, Sri Venkateswara Institute Of Medical Sciences, Tirupati, Andhra Pradesh, India

**Keywords:** Social Media, Urology, Review [Publication Type]

## INTRODUCTION

Social media (SoMe) are changing the way people communicate, interact, and exchange information. Medical and scientific communities are increasingly utilising of these emerging communication tools. Knowledge and scientific content are now broadly available in multiple platforms that allow providers to interact with peers around the world.

These platforms are now facilitating medical education and helping phisicians to increase their network (1). Nowadays, Twitter has played a key role in the medical comunity. It has been shown that Twitter activity can predict articles that will be highly cited (2).

Over 80% of physicians across specialities have some form of social media presence. The current and upcoming generations of physicians entering work force with an innate and natural drive to communicate, this trend is only going to increase (3).

## SCIENTIFIC ASPECTS OF SOCIAL MEDIA

Much has been said about the virtues of social media on popular platforms like Twitter, Facebook, Instagram, etc. Social Media has become a resourceful technology among professionals for the advancement of career as well as staying up-to-date with the latest literature. The internet has brought countless tools to our phones – at the speed of thought. Data has become the most powerful resource and new ideas are the currency of the present. Data can buy robotics and machines and hence, information is to the digital economy what natural oil was to the industrial economy. Scientific analysis of the information pattern shared on social media platforms is overdue -pertaining to quantum of physician usage, reliability of information, index, effect of social media on the impact factor of a journal, etc.

Loeb et al. reported that the most commonly used social media platforms by urologists were Facebook (93%), followed by LinkedIn (46%), Twitter (36%) and Google+ (26%). Physicians less than 40 years was an important predictor of higher social media use (83% vs 56%), with greater uptake among residents/fellows compared to attendings (86% vs 66%). Only 28% of respondents used social media partly or entirely for professional purposes (4). Juan Gomez in his commentary in 2016 quoted that “Twitter is perhaps the social media platform with the most dissemination in healthcare consisting of the broadest possible opportunities for interesting news, knowledge sharing, and networking amongst health professionals” (5, 6). Twitter has 326 million users with 500 million tweets every day. Apart from Twitter, urologys is also the first specialty to use the VineTM platform as a medical education tool and the PeriscopeTM for streaming sessions from a medical conference.

A study on the effect of social media on impact factor of pediatric urology journals using Filtered Journal Citation Reports over a period of 4 years revealed some interesting findings. The presence of a Twitter feed was statistically significant for an increase in impact factor over 4 years (P = 0.017), demonstrating that presence of an article in social media is associated with a rise in the journal impact factor (7).

A study examining 33 prominent urological journals revealed that eight journals had Twitter profiles with a mean of 1845 followers each, ultimately translating into a higher journal impact factor. Journals with a Facebook page were also found to be more likely to have a twitter page (8). In an analysis of 710 articles being shared, and their associated tweets over a six-month period, it was noted that out of the 710 articles 21% were Level 1 evidence-based articles, 14% were level 2, 39% were level 3 and level 25% were level 4.. This adequately gives bearing to the fact that social media can be scientifically accurate in propagation and adsorption of knowledge.

It is pertinent to note that social media articles in urology with a significant number of citations will influence and produce noticeable improvement of the journal impact factor, about 65% of citations were from non-urological literature (8).

## PHYSICIAN ASPECTS

In the current era, social media has evolved into a digital space for networking and learning. This inclination towards networking helps us to participate in conversations with diverse groups of individuals, scientists and intellectuals. It can help provide professionals with reliable and relevant information more effectively than traditional methods like emails and journals. The current technology also gives us an important advantage in connecting with our colleagues and peers from other disciplines on social media. Complex cases can be discussed online and this activity can further promote clinical competence in the management of such cases. The use of social media in conferences has also enriched this interactive aspect. It has allowed vibrant exchange of ideas and information during conferences. It is a unique platform that allows interactions between people regardless of geographical restrictions.

The virtual engagement among physicians also leads to exchange of ideas and collaborations. In the current age of multi institutional studies and collaborative clinical trials it is worthwhile to note that social media has brought about breakthroughs in study recruitment, delivery of interventions and data collection (9). Some aspects of clinical activity in studies designs which were done traditionally in the past with face to face approaches are currently being replaced by social media. A prime example of physicians leveraging social media for research is the REMOTE trial for overactive bladder (10). In this study, the authors Orri et al utilized social media, forums and online websites to recruit and manage participants. This trial has shown the advantages of adopting social media with, improved efficiency and lowered costs in this example. Not only does it help in crowd sourcing, the use of social media in clinical trials also helped to achieve higher follow-up rates (11).

One of the forerunners that brought about this vibrant exchange on social media are the international social media consortiums such as journal clubs and groups (12). These social media platforms have demonstrated value to the surgical community. Labelling of content with “hashtags” used on these platforms allows users to further filter data according to their needs. The concerns of the doctors such as ease of content access is now a thing of the past. Most importantly, these discussions on social media have acted as a podium in seeking timely advise o as well as discussing professional challenges in dealing with clinical and non clinical aspects of healthcare (13).

Education and knowledge update is also an aspect of social media usage, and surgeons are utilizing social media for professional development. One of the foremost modern theories that supports this platform for physician training is the Eraut learning cycle. Social media learning accounts for the informal learning cycle of Eraut. It is the only technology that contributes to implicit, reactive and deliberative learning of the cycle. With duty hours and heavy clinical work, we have fewer opportunities for traditional ward-based learning. Healthcare professionals are increasingly using online educational resources and open access formats available to engage in educational activities. Availability of surgical content online has taken the learning experience one step further and this has even been extended to online learning of surgical skills.

The main reasons for physicians to join social media are well documented. In addition to networking, engaging in medical education, physician branding also plays an important role. Having an online presence has been shown to directly impact clinical practice. With the current set of guidance and amount of work that went into online professionalism, it should not be seen as a challenge but rather an opportunity to both improve and accommodate the traditional values of medicine to the characteristics of social media (14). We should recognize that in this time of translational medicine we need physicians who can connect with stakeholders, improve policy, change clinical practice and social media is one of the best platforms to accomplish this.

## PATIENTS ASPECTS

The use of social media has become very instinctive to many and it has become part of a daily routine. Enhanced communication, liberated expressions of oneself, keeping updated with all the trends and news, marketing and promotion are only some of the reasons why people use social media. Physicians are in the “get people better” business and therefore it is important to develop a strategy on how to use social media to engage and connect with patients. Doing this, it is likely that it will improve the chances of providing a better health care experience.

Patients use social media intended to meet an unfulfilled need. The most common reason for social media use by patients health is for social and emotional support. Social support is defined as “the process of interaction in relationships which is intended to improve coping, esteem, belonging, and competence through actual or perceived exchanges of psychosocial resources”.

A type of social media use by patients is emotional support, that is defined as “communication that meets an individual’s emotional or affective needs”. It refers to support gained through expressions of care and concern, which serve to improve an individual’s mood. Emotional support helps patients to meet their emotional or affective needs. Examples of emotional support are “sharing of emotional difficulties”, “encountering support that feels like a warm blanket wrapped around you”, and “share emotions with other people who are coping with similar problems” (15).

Also, esteem support is a social media use for patients, that refers to “communication that bolsters an individual’s self-esteem or beliefs in their ability to handle a problem or perform a needed task”. The aim of this type of support is to encourage individuals to take the actions needed to successfully live with their condition. Examples of esteem support include “getting support from other patient’s encouragement”, “share experiences about a new treatment to find encouragement before starting it”, and “rituals of confirming each other’ s endeavors to follow health instructions”.

Another patient’s use of use social media is for information gathering. Patients who are newly diagnose with certain conditions are in a need for information about their health conditions and treatment options. These can be found on health care related websites but also on support groups from patients who have already dealt with the particular condition.

## CONCLUSIONS

Social media (SoMe) is a communication revolution and has a great potential to improve health outcomes, although careful and thoughtful utilization is needed to do this effectively. With the continuous increase of social medial use by health care organizations and patients in the daily practice, the risks and benefits of such use needs to be more broadly discussed. Further research is needed to evaluate how twe can incorporate social media into health care in order to improve patients outcomes in the future.
